# Pacemaker implantation in a challenging anatomy: isolated persistent left superior vena cava and azygos continuation of interrupted inferior vena cava

**DOI:** 10.1002/ccr3.1366

**Published:** 2018-03-01

**Authors:** Sergio Conti, Yaariv Khaykin

**Affiliations:** ^1^ Southlake Regional Health Centre Newmarket Ontario Canada

**Keywords:** Azygos vein, coronary sinus anomaly, inferior vena cava, pacemaker, persistent left superior vena cava

## Abstract

Isolated persistent left superior vena cava (SVC) in the absence of right SVC is a rare congenital variant of thoracic venous drainage with the left subclavian and jugular veins that drain into the right atrium through the coronary sinus. Inferior vena cava interruption with azygos continuation is another congenital anomaly resulting in venous drainage of the lower extremities via a typically dilated azygos vein. Although both variants are generally asymptomatic and incidentally detected, these can have clinical implications in specific circumstances and in particular during device implantation. We report a case of pacemaker implantation in which both anatomical variants were present.

A 65‐year‐old man with complete heart block and symptomatic bradycardia presented for a dual‐chamber pacemaker (PM) implantation. His past medical history included hypertension, dyslipidemia, type 2 diabetes, hypothyroidism, and a previous transient ischemic attack. On admission, his blood pressure was 160/70 mmHg with a 12 lead ECG showing sinus rhythm with complete atrioventricular block and a regular wide QRS escape rate of 35 b/m. Laboratory investigations were unremarkable. Chest X‐ray demonstrated an enlarged cardiac silhouette but normal lung and mediastinal structures. Transthoracic echocardiogram did not show significant abnormalities of the cardiac chambers. Procedure was performed in fasting state and in the usual sterile fashion. After left infraclavicular incision was extended to the pectoralis fascia, vascular access to the extrathoracic axillary vein was established using fluoroscopically guided first rib approach. It was impossible to advance a guidewire into the normal course of the superior vena cava (SVC) with the wire tracking from the right into the left subclavian vein. Venography of the left subclavian vein was performed showing the presence of persistent left SVC (P‐LSVC) with dilated coronary sinus (CS) (Fig. 1, panel A, B, C). Interestingly, the P‐LSVC communicated with an enlarged azygous vein (AzV) system that was the “preferential route” for the PM lead making it very difficult to manipulate and advance the lead into the course of the P‐LSVC (Fig. 1, panel D). Through a long sheath inserted through the P‐LSVC into the AzV, contrast was injected demonstrating an interrupted inferior vena cava (IVC) draining into the AzV (Fig. 1, panel E). A right subclavian venography was also performed demonstrating the absence of the right SVC (RSVC) (Fig. 1, panel F). A steerable diagnostic electrophysiology catheter was used to guide the long sheath into the P‐LSVC (Marinr, Medtronic, Minneapolis, MN). Once the P‐LSVC was instrumented with the long sheath, it was possible to guide the PM leads into the lumen of the P‐LSVC and subsequently through the CS and into the right atrium (RA). The ventricular lead was pushed into the right ventricle (RV) by making a loop against the lateral wall of the RA. The stylet was U‐shaped in order to cross the tricuspid valve. The catheter was then advanced into the RV and placed into the midinterventricular septum. A bipolar active fixation ventricular lead was placed at the interventricular septum, and a bipolar active fixation atrial lead was placed at the high RA. The parameter of the leads was as follows: atrial lead: 1.75V at 0.4 msec and ventricular lead: 0.5V at 0.4 msec. Subsequently, a dual‐chamber PM (Medtronic was uneventfully implanted (Fig. 2).

**Figure 1:**
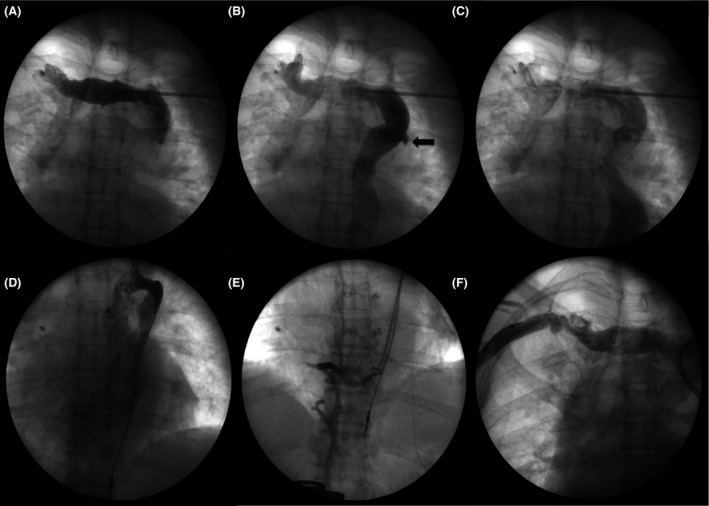
Venography of the left subclavian vein shows the presence of persistent left SVC (Panel A, B, C). PM lead follows the course of the azygous vein (Panel D). Contrast injection through a long sheath inserted in the P‐LSVC demonstrating an IVC draining into the azygos vein (Panel E). Right subclavian vein venography showing the absence of right SVC (Panel F).

**Figure 2:**
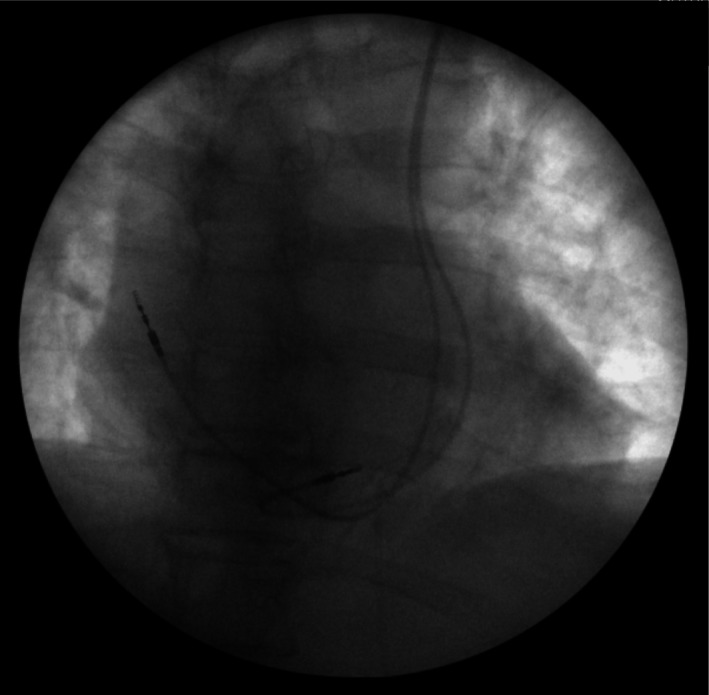
Anteroposterior projection: final leads position. The right atrium lead is positioned into the high right atrium, and the ventricular lead is positioned into the midinterventricular septum.

## Discussion

Presence of P‐LSVC is the most common congenital variant of thoracic venous drainage. It results from the nonregression of the left common cardinal vein and drains the left subclavian and jugular veins into the RA through an enlarged CS. This condition can be isolated and totally asymptomatic, reported in about 0.5% of the general population and generally as an incidental finding on echocardiography or cross‐sectional imaging with computed tomography (CT) with no significant functional importance. It can also be associated with congenital heart disease (i.e., atrial and ventricular septal defects, endocardial cushion defects, Tetralogy of Fallot, cor triatriatum, pulmonary stenosis) and in this case can be present in up to 10% of these patients 1. Isolated P‐LSVC with an absent RSVC is a rare anatomical variation, with the incidence of 0.07–0.13% 2. It has been also associated with an increased risk of developing atrial fibrillation 3. Congenital anomalies of the IVC and its tributaries are also increasingly recognized in asymptomatic patients during a cross‐sectional imaging and CT. The majorities of IVC anomalies are asymptomatic but occasionally present clinically with thromboembolic complications. Azygos continuation of an interrupted IVC can be isolated or occasionally associated with congenital heart disease and has an incidence <1% resulting in venous drainage of the lower extremities in a compensatory enlarged AzV 4.

In the majority of the P‐LSVC cases, during device implantation, access is switched to the right subclavian vein, allowing for an easier route for lead manipulation. However, in this case, it was not a viable option due to the absence of RSVC. In addition, interrupted IVC draining into the AzV further complicated lead manipulation and navigation. This combination of anatomical variants also precludes the possibility of leadless pacemaker implantation. To the best of our knowledge, association of isolated P‐LSVC and azygos continuation of interrupted IVC has never been previously reported in patients undergoing PM implantation. These challenging anatomical variants, often discovered at the time of the procedure, can pose difficulties and complications during central venous cannulation or device implantation such as arrhythmia, cardiogenic shock, cardiac tamponade, CS dissection, and thrombosis. However, improvements in materials and techniques over years allowed the successful implantation of cardiac rhythm management devices including pacemakers, implantable cardioverter‐defibrillators, and cardiac resynchronization therapy devices. It is important to be aware of the anatomical variants during device implantation procedures in order to successfully overcome these challenges.

## Authorship

SC: wrote the case report. YK: was the operator who performed the procedure.

## Conflict of Interest

None declared.
